# Detection and perception of generic host volatiles by mosquitoes modulate host preference: context dependence of (*R*)-1-octen-3-ol

**DOI:** 10.1098/rsos.160467

**Published:** 2016-11-09

**Authors:** Shahid Majeed, Sharon Rose Hill, Göran Birgersson, Rickard Ignell

**Affiliations:** Unit of Chemical Ecology, Department of Plant Protection Biology, Swedish University of Agricultural Sciences, PO Box 102, SE-230 53 Alnarp, Sweden

**Keywords:** (*R*)-1-octen-3-ol, chemical analysis, behaviour, electrophysiology, *Anopheles coluzzii*, *Aedes aegypti* and *Culex quinquefasciatus*

## Abstract

Natural selection favours a restricted host breadth in disease vector mosquitoes, indicating that there is an adaptive value associated with maintaining plasticity in host preference. One mechanism to maintain such plasticity is via the detection of generic cues by conserved peripheral olfactory pathways, which when perceived in different host odour contexts enable the identification of and discrimination among potential host species. Here, we show that the context of an odour cue shapes host perception in mosquitoes, by altering the release rate of the generic host-related volatile (*R*)-1-octen-3-ol, within its natural range, and in the background odour of known hosts and non-hosts. This result highlights that host recognition is contextual and dependent on quantitative and qualitative differences in odour blends and the olfactory codes evoked. From the perspective of vector management, understanding the perception of odour blends and their context is essential to the process of developing synthetic blends for the optimal attraction of mosquitoes in efforts to control and monitor populations.

## 1. Background

Inherent host preference is often a marked characteristic of mosquito disease vectors [1–3], and while blood-feeding preference is inherent in mosquitoes [1,3], it can be modulated by host availability [4,5]. The plasticity of host preference, a key trait regulating disease transmission by anthropophilic mosquitoes [6], underlies the ability of mosquitoes to adapt to varying ecological conditions [1,3,5]. Natural selection on the malaria vectors *Anopheles gambiae sensu lato,* and the arbovirus vectors *Aedes aegypti* and *Culex quinquefasciatus* has, however, been shown to favour a restricted host breadth [3], indicating an adaptive value to maintaining plasticity [1]. We hypothesize that such plasticity could be maintained through the use of generic cues, which are common to many host species and detected by conserved olfactory receptor neuron pathways, placed in different host odour contexts to identify and discriminate among potential host species.

Mosquitoes locate their hosts primarily through olfaction [3,7]. A sequence of behaviours contribute to host discrimination and selection, including activation, long- and short-range attraction, and landing on the host [8]. Minute fluctuations in carbon dioxide (CO_2_) concentration elicit activation and attraction in host-seeking mosquitoes, which constitutes the initial recognition of a potential host [8–11]. Host odours take on a more prominent role in short-range attraction and landing [8,12]. Close to the host, mosquito behavioural responses to complex host odours are more robust than to single host volatiles, indicating that volatile blends play a crucial role in the coding for host odour recognition [7,13–15]. Although species-specific host volatiles may be involved in host discrimination and selection by anthropophilic mosquitoes [16,17], an increasing body of research suggests that host perception relies on a number of generic host volatiles and their relative proportions [7,9,18–20]. To process qualitative and quantitative differences among host blends, mosquitoes use coincidence detection to distinguish among hosts [7], similar to what has been shown for herbivorous insects [21]. Such coincidence detection has been shown to be essential for the behavioural response to one of the best-characterized generic host-related volatiles, (*R*)-1-octen-3-ol [7,22,23]. While not active by itself, (*R*)-1-octen-3-ol requires the simultaneous detection of CO_2_ to elicit behavioural attraction [22]. This has, however, only been shown for release rates that exceed the natural emission of (*R*)-1-octen-3-ol from known hosts [22]. The advantage of coincidence detection is that it is a flexible odour coding system, which allows for adaptation to alternative host species [21].

In this study, we investigate how the context of an odour cue shapes host perception in mosquitoes. We demonstrate that an individual host compound is interpreted in the context of the host odour. By altering the release rate of the generic host-related volatile (*R*)-1-octen-3-ol within its natural range, we demonstrate that blend context plays an important role in host and non-host recognition.

## 2. Material and methods

### 2.1. Insects

*Aedes aegypti* (Rockefeller strain), *An. coluzzii* (Suakoko strain) and *C. quinquefasciatus* (Thai strain) were reared at 27 ± 2°C, 70 ± 2% relative humidity (RH) under a 12 L : 12 D period, as previously described [22,24]. For all experiments, 4- to 10-day post-emergence sugar-fed adult female mosquitoes were used.

### 2.2. Volatile collections

Headspace volatile extracts were collected from humans, chickens and cattle hair. Human body volatiles were collected as previously described [25], with minor modifications. Briefly, naked volunteers were placed in customized heat-sealed bags (2 × 1.75 m; Melitta, Helsingborg, Sweden), with only their heads protruding. Empty bags of the same size were used as controls. Synthetic air (20.9% O_2_ and 79.1% N_2_, Strandmöllen AB, Ljungby, Sweden) was introduced into the bags at a rate of 6.5 l min^−1^. Pumps (reversed aquarium pumps; Rena 301, Rena, USA) extracted the air at 0.9 l min^−1^ through seven columns containing 40 mg Porapak Q (PQ; 80/100 mesh, Alltech, Deerfield, IL, USA) over 2.5 h. Volatiles were collected from a live chicken (Gammalsvensk dvärghöna) placed on a metal mesh in a desiccator covered with a black cloth to keep it calm. Charcoal filtered air was introduced (1 l min^−1^) via a Teflon tube and pumped out of the desiccator via a glass splitter connected to four PQ adsorbent columns (0.25 l min^−1^ each) over 1 h. Volatiles from Holstein cattle were collected by placing 20 g of freshly cut hair, approximately one third of the hair on a single cow, in a glass wash bottle. Charcoal filtered air was drawn by pumps (0.1 l min^−1^) through the bottle onto a PQ adsorbent column over 24 h. Trapped volatiles were desorbed by eluting each column with 600 µl of pentane (*puriss p.a.*, Sigma-Aldrich Chemie GmbH, Steinheim, Germany). The volatile collections from each group and species were pooled and concentrated under a gentle stream of nitrogen to contain 0.25 min equivalents µl^−1^ for use in further experiments. Before use, the adsorbent columns were rinsed with 1 ml each of methanol, dichloromethane and pentane. Heptyl acetate (1 µg, 99.8%; Aldrich, St Louis, MO, USA) was added to each extract as an internal quantification standard before concentration.

### 2.3. Single sensillum recordings

The maxillary palps of *Ae. aegypti*, *An. coluzzii* and *C. quinquefasciatus* are covered with capitate peg sensilla, variously described as peg sensilla or basiconic sensilla, each housing three olfactory receptor neurons [22,26–28]. In all species, the intermediate amplitude neuron, referred to as the B cell, has been shown to respond to (*R*)-1-octen-3-ol [22,27,28]. Electrophysiological recordings from the B cells were made as previously described [22].

### 2.4. Stimulation and stimuli

An Agilent 6890 gas chromatograph (GC; Agilent Technology, Santa Clara, CA, USA) fitted with a fused silica capillary column (30 m × 0.25 mm i.d.) coated with non-polar HP-5 stationary phase (d.f. = 0.25 µm) was used for the separation of volatiles in the collected extracts, with hydrogen gas as the mobile phase (*Q* = 45 cm s^−1^). Aliquots of each sample (2 µl) were injected splitless for 30 s at 225°C. The GC oven temperature was programmed from 30°C (3 min hold), followed by a ramp of 8°C min^–1^ to 225°C, and held isothermal for 10 min. The GC was fitted with a make-up gas-fed (4 psi N_2_) four-way cross (Graphpack® 3D/2 Crosspiece Sulfinert™, Gerstel, Mülheim an der Ruhr, Germany) at the end of the column, delivering half of the effluent to the flame ionization detector, and the other half to the air stream passing over the maxillary palp via a Gerstel ODP-2 transfer line maintained at 135°C for 15 min and increased at 8°C min^–1^. Bioactive compounds were identified by injection on a combined Agilent 6890N GC and 5975 mass spectrometer (GC-MS; Agilent Technology) fitted with an HP-5MS column (dimensions as above), and using the same programme as for the GC-SSR analyses, but with helium as the mobile phase (*Q* = 35 cm s^−1^). The active compounds were identified by comparison with reference mass spectra in our custom-made and commercially available libraries (NIST05 and Wiley). Identified compounds were confirmed by parallel injections of synthetic reference compounds with authentic samples on the GC-MS. In addition, the bioactive compound (*R*)-1-octen-3-ol was quantified by the extracted ion current profile of *m*/*z* 57 [29] in the human extract, and in the cattle extract, by ratio compared with the internal standard. For verification of bioactive amounts, synthetic (*R*)-1-octen-3-ol (99%; courtesy of Dr James Logan) [22] dissolved in redistilled hexane was injected into the GC-SSR at concentrations ranging from 0.001 to 100 ng µl^−1^.

### 2.5. Landing bioassay

Behavioural responses of mosquitoes to (*R*)-1-octen-3-ol were observed in a no-choice landing assay by using a membrane feeding apparatus (Discovery Workshops, Accrington, Lancashire, UK) as previously described [30]. Volatile extracts (10 µl; 2.5 min equivalents) were applied on a collagen membrane, and regulated at 37°C for *Ae. aegypti* and *An. coluzzii*, and 42°C for *C. quinquefasciatus*. Experiments were conducted at 27 ± 2°C and 68 ± 2% RH, under either white or red light. Eight treatments (*n* = 10 for each species) were tested: (i) human hand rubbing for 1 min; (ii) cattle hair extract; (iii) chicken extract; (iv) 0.1 ng of (*R*)-1-octen-3-ol; (v) 5 ng of (*R*)-1-octen-3-ol; (vi) human hand rubbing with a cattle equivalent of (*R*)-1-octen-3-ol (5 ng); (vii) chicken extract with a human equivalent of (*R*)-1-octen-3-ol (0.1 ng); and (viii) hexane (control). For all treatments, the total volume added to the membrane was 20 µl, and where this was not provided by the treatments themselves, the remainder was supplied by the solvent, hexane. Initial control tests verified that human hand rubbing was not significantly different (ANOVA, *F* = 0.06, d.f. = 1, *p* = 0.80) from the human body extract (10 µl; 2.5 min equivalents) in a two-choice assay (data not shown).

Twenty individual mosquitoes were kept in an experimental cage (30 × 30 × 30 cm; Bugdorm, MegaView Science, Taiwan) under experimental climatic conditions for 24 h prior to the experiment. The number of landing mosquitoes was observed every 30 s for 10 min. Pure CO_2_ was pulsed into the cage for 4 s, once every minute (0.2 l min^−1^). The numbers of mosquitoes landing every 30 s reached equilibrium after 6 min, and the number of mosquitoes landing after 10 min was used for all further comparisons. Experiments were performed in the last 4 h of photophase for *Ae. aegypti* and during scotophase for *An. coluzzii* and *C. quinquefasciatus*.

### 2.6. Statistical analysis

Repeated measures two-way ANOVA, followed by a Bonferroni *post hoc* test was performed to compare the physiological activity among the species. A general linear model (GLM) two-way ANOVA, followed by Bonferroni *post hoc* test was used to calculate the significance within the eight different treatments of each species in the landing bioassay. The repeated measures two-way ANOVA was carried out with Graph Pad Prism v. 5.01 for Mac (GraphPad Software, La Jolla, CA, USA), while the GLM two-way ANOVA was analysed using Minitab v. 16.1.0 (Minitab Statistical Software, State College, PA, USA).

## 3. Results

### 3.1. Detection of the natural emission rates of (*R*)-1-octen-3-ol

**Figure 1.**
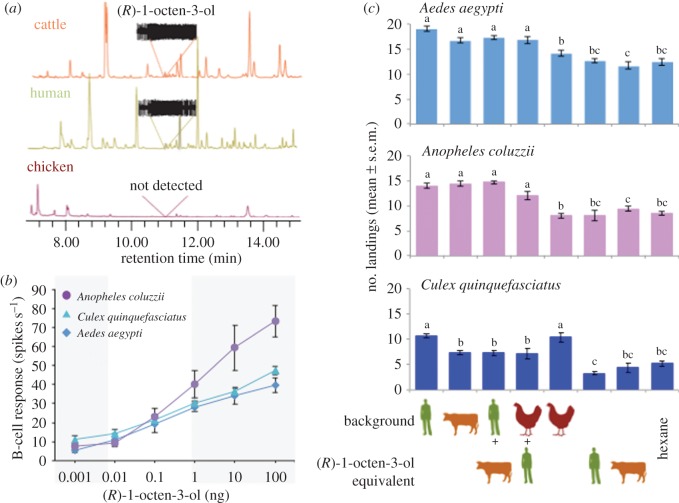
Females of *Aedes aegypti, Anopheles coluzzii* and *Culex quinquefasciatus* respond physiologically and behaviourally to (*R*)-1-octen-3-ol. (*a*) Representative odourant profiles of cattle (orange), human (green) and chicken (dark red) extracts as generated by gas chromatography (GC). (*R*)-1-octen-3-ol was present in the cattle and human extracts but was not detected (nd) in the chicken extracts. Traces above the human and cattle extract represent the elicited responses from the (*R*)-1-octen-3-ol sensitive neuron (the B cell) in *Ae. aegypti*, using GC-single sensillum recordings (SSR). (*b*) Averaged GC-SSRs from *Ae. aegypti* (light blue), *Cu. quinquefasciatus* (dark blue) and *An. coluzzii* (purple) to synthetic (*R*)-1-octen-3-ol (*n* = 5) show a dose-dependent relationship. The white panel indicates the range of natural release of (*R*)-1-octen-3-ol from human and cattle. (*c*) Landing bioassays were performed to observe the role of (*R*)-1-octen-3-ol in host-seeking behaviour (*n* = 10). The different extracts were tested alone and in combination with human and cattle equivalents of (*R*)-1-octen-3-ol (0.1 and 5 ng over 10 min, respectively). Human and cattle equivalents of (*R*)-1-octen-3-ol were also tested individually, as was the solvent hexane. Letters above the bars denote significant difference between treatments within species (GLM ANOVA, *p* < 0.05).

The GC-SSR (figure 1*a*) and GC-MS analyses identified (*R*)-1-octen-3-ol as a key natural ligand for one of the olfactory receptor neurons of the capitate peg sensillum, the B cell. (*R*)-1-octen-3-ol was present in the cattle hair (0.6 ± 0.35 ng min^−1^ equivalent) and human extracts (0.06 ± 0.04 ng min^−1^ equivalent), but was not detected in the chicken extract (figure 1*a*). Considering the difference in the methods used to collect the headspace of cattle (shaved hair), compared to human and chicken (whole body), we may be underestimating the release rate of (*R*)-1-octen-3-ol for the cow. Dose–response analysis, using GC-SSRs, revealed a similar B cell response among the three mosquito species to (*R*)-1-octen-3-ol (*F* = 3.522, d.f. = 2, *p* = 0.063). The response to (*R*)-1-octen-3-ol that approximates the human release rate (0.01–0.1 ng) reflects the threshold of detection for the B cell in these mosquitoes, while that associated with cattle hair (0.5–1.0 ng) is well within the detection range of the OSN (*F* = 86.98, d.f. = 5, *p* < 0.001; figure 1*b*; white panel).

### 3.2. Behavioural response to (*R*)-1-octen-3-ol

The landing behaviour of mosquitoes was examined in the presence of different odour extracts. *Aedes aegypti* and *An. coluzzii* landed significantly more often on the human hand rubbing and the cattle hair extract than on both the chicken extract and the control (*F* = 30.85, d.f. = 3, *p* < 0.001; *F* = 52.82, d.f. = 3, *p* < 0.001; figure 1*c*). In both species, addition of a human equivalent (0.1 ng) of (*R*)-1-octen-3-ol to chicken extract increased landing to a level comparable to the human and cattle extracts (*F* = 8.09, d.f. = 3, *p* < 0.001; *F* = 25.22, d.f. = 3, *p* < 0.001). Human hand rubbing with a cattle equivalent (5 ng) of (*R*)-1-octen-3-ol, however, did not increase the landing above that of the cattle extract alone (*F* = 1.24, d.f. = 1, *p* < 0.281; *F* = 0.23, d.f. = 1, *p* < 0.634; figure 1*c*).

*Culex quinquefasciatus* showed similar landing responses in the presence of the human hand rubbing and chicken extracts, while the behaviour towards the cattle extract and the extracts (human and chicken) with added (*R*)-1-octen-3-ol did not differ significantly from the control (*F* = 1.64, d.f. = 3, *p* < 0.22; figure 1*c*). Interestingly, when the human equivalent of (*R*)-1-octen-3-ol was added to the chicken extract, a reduction in landing was observed compared with chicken extracts alone (*F* = 17.06, d.f. = 7, *p* < 0.001; figure 1*c*). The control and (*R*)-1-octen-3-ol alone (0.1 and 5 ng) were not significantly different from each other in all species (*F* = 0.70, d.f. = 2, *p* < 0.504; *F* = 1.04, d.f. = 2, *p* < 0.368; *F* = 1.32, d.f. = 2, *p* < 0.284; figure 1*c*). A two-choice assay verified that human body extract was not significantly different from hand rubbing (*F* = 0.06, d.f. = 1, *p* < 0.80).

## 4. Discussion

The mechanism by which the breadth of mosquito host choice is regulated is a product of the combination of sensory adaptation to preferred hosts over evolutionary time and the phenotypic plasticity of the sensory response [7,31]. Here, we demonstrate that the detection of (*R*)-1-octen-3-ol by the olfactory system and its perception within a blend plays a vital role in host selection and discrimination. Odour differences among hosts, both qualitative and quantitative, significantly affect mosquito behavioural output, highlighting that accurate host recognition results from the perception of generic volatiles in the context of the host odour blend. The data provided here, and in our companion paper [11], emphasize that generic host volatiles provide host recognition cues for mosquitoes, and that the detection and perception of such volatiles provide mosquitoes with a flexible, yet constrained, coding system for host finding.

### 4.1. Detection of (*R*)-1-octen-3-ol at natural release rates

The peripheral olfactory system is conserved across species and is detecting (*R*)-1-octen-3-ol within the ecologically relevant release rates of the compound by potential mammalian hosts. The fact that (*R*)-1-octen-3-ol was not detected in the chicken extracts is in line with previous studies on chickens and other bird species [32–34]. The observed increase in sensitivity of *An. coluzzii* B cells to the highest two concentrations tested may be related to the combination of the increased transcript abundance and sensitivity of its cognate odorant receptor, AgOr8 [27,35], compared with both *Ae. aegypti* (AaOr8 [36,37]) and *C. quinquefasciatus* (CqOr118 and CqOr113 [38]). Any observed differential behaviours in response to the natural release rates of (*R*)-1-octen-3-ol are thus not linked to differential peripheral coding, but are more likely to be a result of combinatorial coding in higher olfactory centres, similar to what has been described in herbivorous insects [21].

### 4.2. Blend perception regulates host choice

The landing response of *Ae. aegypti*, *An. coluzzii* and *C. quinquefasciatus* to the natural headspace volatiles of the preferred and non-preferred host species, but not to the single components therein, underlines that individual components of a host blend, such as (*R*)-1-octen-3-ol (this study), are often not recognized as a host cue when perceived outside the context of the blend [21]. Insects rely on ratio and coincidence detection by olfactory receptor neurons tuned to generic and host-specific volatiles [21]. Such an odour coding system provides flexibility to accommodate for variation in the ratio of volatiles within preferred host blends. For instance, *Ae. aegypti* and *An. coluzzii* continue to land following the increase in the ratio of (*R*)-1-octen-3-ol in human headspace to the cattle equivalent. However, *C. quinquefasciatus* did not respond to the headspace of their preferred hosts, human and chicken, after supplementation with cattle or human equivalent doses of (*R*)-1-octen-3-ol, respectively. Conversely, *Ae. aegypti* and *An. coluzzii* responded to the headspace of their non-preferred host, chicken, after supplementation with a human equivalent dose of (*R*)-1-octen-3-ol. Combined, these results emphasize that host recognition is contextual and depends on quantitative and qualitative differences in blends and the olfactory codes they evoke. Similar patterns have been observed in studies on herbivorous insects [21], suggesting that the ratios of host volatiles play a key role in blend perception and host recognition.

## 5. Conclusion

While the role of generic host volatiles in species-specific, particularly human, host selection by mosquitoes has been indicated in previous work [3,7,11], this study highlights the importance of generic host volatiles, within their natural release rates, in modulating interspecific host recognition. The cross-species comparison revealed the importance of analysing the response properties and tuning of olfactory receptor neurons together with how this affects the behavioural output. The study also emphasized the importance of analysing the behavioural response to generic host volatiles in the context of the host blend. From a vector control perspective, this is essential when developing synthetic blends for optimal attraction of mosquitoes in efforts to control and monitor populations.

## Data accessibility

All data that is relevant to this study is reported within the article.
